# Sex-dependent calcium hyperactivity due to lysosomal-related dysfunction in astrocytes from *APOE4* versus *APOE3* gene targeted replacement mice

**DOI:** 10.1186/s13024-020-00382-8

**Published:** 2020-06-09

**Authors:** Raquel Larramona-Arcas, Candela González-Arias, Gertrudis Perea, Antonia Gutiérrez, Javier Vitorica, Tamara García-Barrera, José Luis Gómez-Ariza, Raquel Pascua-Maestro, María Dolores Ganfornina, Eleanna Kara, Eloise Hudry, Marta Martinez-Vicente, Miquel Vila, Elena Galea, Roser Masgrau

**Affiliations:** 1 Unitat de Bioquímica de Medicina, Departament de Bioquímica i Biologia Molecular, and, Institut de Neurociències (INc), Facultat de Medicina, Universitat Autònoma de Barcelona, 08193 Cerdanyola del Vallès, Barcelona, Catalonia Spain; 2 Cajal Institute, Consejo Superior de Investigaciones Científicas (CSIC), 28002 Madrid, Spain; 3 Departamento de Biología Celular, Genética y Fisiología, Facultad de Ciencias, Instituto de Investigación Biomedica de Málaga (IBIMA), Universidad de Málaga, 29071 Málaga, Spain; 4 Centro de Investigación Biomédica en Red sobre Enfermedades Neurodegenerativas (CIBERNED), 28031 Madrid, Spain; 5 Departamento de Bioquímica y Biología Molecular, Facultad de Farmacia, Universidad de Sevilla, Instituto de Biomedicina de Sevilla (IBiS)-Hospital Universitario Virgen del Rocío/CSIC/Universidad de Sevilla, 41012 Sevilla, Spain; 6 Departamento de Química, Facultad de Ciencias Experimentales, Campus de El Carmen, Centro de Investigación en Recursos Naturales, Salud y Medio Ambiente (RENSMA), Universidad de Huelva, 21007 Huelva, Spain; 7 Departamento de Bioquímica y Biología Molecular y Fisiología, Instituto de Biología y Genética Molecular, Universidad de Valladolid-CSIC, 43007 Valladolid, Spain; 8 Alzheimer’s Disease Research Laboratory, MassGeneral Institute for Neurodegenerative Disease, Massachusetts General Hospital, Harvard Medical School, Charlestown, MA 02129 USA; 9 Present Address: Institute of Neuropathology, University Hospital of Zurich, 8091 Zurich, Switzerland; 10 Neurodegenerative Diseases Research Group, Vall d’Hebron Research Institute (VHIR), 08035 Barcelona, Spain; 11 ICREA, Passeig Lluís Companys 23, 08010 Barcelona, Catalonia Spain

**Keywords:** *APOE4*, Astrocytes, Calcium signaling, Sex, Lysosome, Purinergic receptors, Lipidome

## Abstract

**Background:**

The apolipoprotein E (*APOE*) gene exists in three isoforms in humans: *APOE2, APOE3* and *APOE4*. *APOE4* causes structural and functional alterations in normal brains, and is the strongest genetic risk factor of the sporadic form of Alzheimer’s disease (LOAD). Research on *APOE4* has mainly focused on the neuronal damage caused by defective cholesterol transport and exacerbated amyloid-β and Tau pathology. The impact of *APOE4* on non-neuronal cell functions has been overlooked. Astrocytes, the main producers of ApoE in the healthy brain, are building blocks of neural circuits, and Ca^2+^ signaling is the basis of their excitability. Because *APOE4* modifies membrane-lipid composition, and lipids regulate Ca^2+^ channels, we determined whether *APOE4* dysregulates Ca^2+^signaling in astrocytes.

**Methods:**

Ca^2+^ signals were recorded in astrocytes in hippocampal slices from *APOE3* and *APOE4* gene targeted replacement male and female mice using Ca^2+^ imaging. Mechanistic analyses were performed in immortalized astrocytes. Ca^2+^ fluxes were examined with pharmacological tools and Ca^2+^ probes. *APOE3* and *APOE4* expression was manipulated with GFP-*APOE* vectors and *APOE* siRNA. Lipidomics of lysosomal and whole-membranes were also performed.

**Results:**

We found potentiation of ATP-elicited Ca^2+^responses in *APOE4* versus *APOE3* astrocytes in male, but not female, mice. The immortalized astrocytes modeled the male response, and showed that Ca^2+^ hyperactivity associated with *APOE4* is caused by dysregulation of Ca^2+^ handling in lysosomal-enriched acidic stores, and is reversed by the expression of *APOE3*, but not of *APOE4*, pointing to loss of function due to *APOE4* malfunction. Moreover, immortalized *APOE4* astrocytes are refractory to control of Ca^2+^ fluxes by extracellular lipids, and present distinct lipid composition in lysosomal and plasma membranes.

**Conclusions:**

Immortalized *APOE4* versus *APOE3* astrocytes present: increased Ca^2+^ excitability due to lysosome dysregulation, altered membrane lipidomes and intracellular cholesterol distribution, and impaired modulation of Ca^2+^ responses upon changes in extracellular lipids. Ca^2+^ hyperactivity associated with *APOE4* is found in astrocytes from male, but not female, targeted replacement mice. The study suggests that, independently of Aβ and Tau pathologies, altered astrocyte excitability might contribute to neural-circuit hyperactivity depending on *APOE* allele, sex and lipids, and supports lysosome-targeted therapies to rescue *APOE4* phenotypes in LOAD.

## Background

Apolipoprotein E is a component of lipoproteins involved in extracellular lipid transport and cholesterol fluxes throughout the body, including the brain [1]. In humans, *APOE* exists in three isoforms: *APOE2*, *3*—the most common allele—and *4*. Although the three isoforms differ only in the amino acids in positions 112 and 158 at the N-terminal domain of the protein [2], this minimal difference results in a structural change in the *APOE4* isoform that profoundly compromises its function [3], as shown by a wealth of studies in mice and humans that document the impact of *APOE* genotype on the structure and function of the healthy brain. Thus, in humans, *APOE4* is associated with reduced memory retention [4], altered neural activity and brain connectivity [5], reduced grid-cell like representations [6], reduced dendritic spine density [7], and hypometabolism measured with fluorodeoxyglucose-based PET [8]. Furthermore, *APOE4* knock-in mice present alterations of behavior, olfactory memory and neurotransmission, as well as decreased dendritic arborization and metabolic alterations [9–13], as compared to *APOE3* knock-in mice. Not only is normal brain function compromised by *APOE4*, but *APOE4* is also the strongest genetic risk factor in late-onset Alzheimer’s disease (LOAD) [14], the principal cause of age-related dementia, affecting millions of people around the world [15]. A complex interaction exists among sex, age and *APOE4* load. Thus, according to a meta-analysis, heterozygous *APOE3*/*APOE4* women present increased risk of LOAD between the ages of 65 and 75 years, and increased risk of mild cognitive impairment (MCI) between the ages of 55 and 70, as compared to men [16]. Homozygous *APOE4* subjects show increased risk compared to *APOE3/APOE4* heterologous individuals [16–18], with men being at greater risk as reviewed by Riedel and colleagues [17] but not according to other authors [18]. In addition, detrimental actions of *APOE4* have been reported in other neurodegenerative disorders such as frontotemporal dementia [19], cerebrovascular disease [20] and traumatic brain injury [21].

The mechanisms whereby *APOE4* is pathological in normal and diseased brain are not totally clarified [15]. An outstanding question is whether *APOE4* affects the function of brain cells other than neurons. Although microglia and neurons secrete ApoE in pathological conditions, as described in mouse neurons injured with kainic acid [22], and in human LOAD brains, where microglia produces and deposits ApoE in senile plaques [23] and appears to mediate Tau pathology [24], it is often overlooked that *APOE* is mainly synthesized, secreted, and lipidated by astrocytes in physiological and pathological conditions [3]. Astrocytes can also take up lipoproteins, as they express *APOE* receptors such as the LRP1 receptor [25].

Astrocytes are building blocks of neural circuits, where they modulate neuronal activity [26]. Apart from the impact of *APOE4* on synaptogenesis [27] and synaptic transmission [28] by lowering the delivery of cholesterol from astrocytes to neurons, the question of whether *APOE4* compromises the global control of astrocytes over neuronal activities has not been examined. Among phenomena modulated by astrocytes there are, to cite a few, long-term potentiation (LTP), memory consolidation, and circadian rhythms [29–32]. Central to astrocyte-to-neuron communication is Ca^2+^ signaling in astrocytes. Thus, although astrocytes are considered non-excitable cells in terms of action potentials, they respond by way of Ca^2+^ signals to neurotransmitters. In turn, such Ca^2+^ responses promote the release of several molecules called gliotransmitters, such as ATP, glutamate, D-serine or GABA [29, 30, 32–34]. Ca^2+^ signaling is thus considered the basis of astrocyte excitability, which is exquisitely precise owing to unique spatiotemporal Ca^2+^ features resulting from the combination of Ca^2+^ signaling pathways orchestrated by different second messengers, and intracellular organelles [35]. Pathways include inositol 1,4,5-triphosphate (IP3)-mediated Ca^2+^ release from the endoplasmic reticulum (ER) [36], and nicotinic acid adenine dinucleotide phosphate (NAADP)-elicited mobilization from acidic organelles, a heterogeneous population of vesicles highly enriched in lysosomes [37]. Extracellular Ca^2+^ entry and mitochondrial Ca^2+^ uptake can further shape cytosolic Ca^2+^ increases [37, 38]. Of note, dysregulation of Ca^2+^ signaling in astrocytes has been reported in animal models of LOAD [39]. However, Ca^2+^ signaling has never been studied in astrocytes in the context of *APOE4*.

*APOE* genotype confers distinct composition to lipid membranes [40], and it is well established that lipid composition (e.g., contents of cholesterol and phospholipids) affects the function of membrane-associated enzymes, receptors, and channels [41]. On the basis of this evidence, we posited that *APOE4* dysregulates Ca^2+^ excitability in astrocytes by modifying membrane lipid composition. To test this hypothesis, we studied ex vivo Ca^2+^ signaling in hippocampal astrocytes of female and male mice with human *APOE3* and *APOE4* gene targeted replacement of the endogenous mouse *APOE* [42, 43]. We found that Ca^2+^ responses induced by the stimulation of purinergic receptors were upregulated in *APOE4* versus (vs) *APOE3* astrocytes in male, but not in female mice. Next, we clarified the underlying mechanisms in cultured immortalized astrocytes expressing human *APOE3* and *APOE4* [44]. The cells reproduced the ex vivo model of male mice, as Ca^2+^ responses were increased in immortalized *APOE4* cells as compared to *APOE3* cells. Cultured *APOE4* astrocytes released more Ca^2+^ from their acidic stores upon purinergic stimulation. Manipulation of *APOE4* and *APOE3* expression revealed that it is the allele, not the reduced synthesis, that caused altered Ca^2+^ signaling in immortalized *APOE4* astrocytes. Finally, we demonstrated that *APOE4* astrocytes have distinct lipidome and were refractory to control Ca^2+^ fluxes by extracellular lipids. Taken together, the data suggest that the *APOE* genotype modulates Ca^2+^ fluxes in astrocytes in a lipid, lysosome and sex-dependent manner. Future research will clarify whether dysregulation of astrocyte excitability contributes to the increased risk of developing LOAD and other brain pathologies in *APOE4* carriers.

## Materials and methods

### Animal model

Nine- to 12-week-old male and female *APOE3* and *APOE4* transgenic mice homozygous for the human *APOE3* or *APOE4* gene replacing the endogenous mouse *APOE* gene were purchased from Taconic (USA) [42, 43]. All experimental procedures were performed according to the animal research regulations (RD53/2013 and 2010/63/UE) of Spain and the European Union, and with the approval of the Committees of Animal Research from the Institutional Animal Ethics Committee of CSIC. Animals were housed in standard laboratory cages with ad libitum access to food and water, under a 12:12 h dark-light cycle in temperature controlled rooms.

### Cell culture

*APOE3* and *APOE4* immortalized astrocytes were a gift from Dr. Holtzman (Washington University) [44]. Immortalized astrocytes were routinely grown at 37 °C in 5% CO_2_ humidified atmosphere air in advanced DMEM medium supplemented with 1 mM of Na^+^ pyruvate, 10% fetal bovine serum (FBS), 100 U/mL of penicillin, 100 μg/mL of streptomycin, and 0.2 mg/ml of geneticine. Cell passages were performed with trypsin once per week up to passage 10.

### Ca^2+^ imaging in astrocytes ex vivo in brain slices

Animals were sacrificed; the brain was rapidly removed and placed in ice-cold modified artificial cerebrospinal fluid medium (ACSF). To reduce swelling and damage in superficial layers, N-methyl-D-glucamine (NMDG)-ACSF was used during brain sectioning, NMDG being a substitute for sodium ions in a wide range of adult ages and applications [45]. NMDG-ACSF contained [in mM]: NMDG 93, KCl 2.5, NaH_2_PO_4_ 1.2, NaHCO_3_ 30, HEPES 20, MgSO_4_ 10, CaCl_2_ 0.5, glucose 25, sodium ascorbate 5, thiourea 2, sodium pyruvate 3, gassed with 95% O_2_/5% CO_2_ (pH 7.3–7.4). Hippocampal slices (350 μm) obtained with a vibratome (Leica Vibratome VT1200S, Germany) were incubated in NMDG-ACSF for 10 min at 34 °C, and then equilibrated for more than 1 h at room temperature (22–24 °C) prior cell loading with fluorochromes in ACSF containing [in mM]: NaCl 124, KCl 2.69, KH_2_PO_4_ 1.25, MgSO_4_ 2, NaHCO_3_ 26, CaCl_2_ 2, and glucose 10, gassed with 95% O_2_/5% CO_2_ (pH 7.3–7.4). Slices were loaded with sulforhodamine 101 (SR101, 1 μM) for 20 min at 34 °C for astrocyte labeling [46–48], and washed in ACSF for 10 min at 34 °C. After SR101 labeling, slices were loaded with 2 mM of fluo-4/AM for 20–30 min at room temperature. After washing fluo-4/AM overloading in ACSF, slices were kept in ACSF medium supplemented with 10% FBS at the stage of a Nikon Eclipse FN1 microscope coupled to a CCD camera (ORCA-R^2^, Hamamatsu, Japan). Cells were illuminated for 100–200 ms at 490 nm using LED system (CoolLED pE-100), and images from stratum radiatum astrocytes were acquired every 1 s. The LED system and the camera were controlled and synchronized by the NIS-Elements software (Nikon, Japan) that was also used for epifluorescence measurements. Astrocyte Ca^2+^ levels were recorded from the astrocyte cell body, and Ca^2+^ variations were quantified as changes in the fluorescence signal (F) over the baseline (F_0_) ((F-F_0_)/F_0_). Two protocols of Ca^2+^ monitoring were used. Firstly, spontaneous events were studied by recording Ca^2+^ events for 120 s. Secondly, ATP-induced responses were studied by recording Ca^2+^ baseline for 30 s, followed by local application of ATP (1 mM; 5 s) to activate purinergic receptors for 30 s, and post ATP period for 60 s. Local application of ATP was delivered by pressure pulses through a micropipette (Picospritzer II, Parker Hannifin, Mayfield Heights, OH, USA) in the presence of tetrodotoxin (TTX, 1 μM). Matlab software (MATLAB R2016; Mathworks Natick) custom-written plugin was used for computation of fluorescence values of each region of interest (ROI).

### Ca^2+^imaging in astrocytes in vitro in immortalized astrocytes

Intracellular Ca^2+^ measurement was performed using 2 μM of fura-2/AM, a ratiometric fluorescence indicator whose fluorescence ratio (R_340/380_) is proportional to Ca^2+^ concentration. Imaging of Ca^2+^ signaling in organelles was performed using genetically encoded Ca^2+^ indicators (GECI). The plasmids for endoplasmic reticulum (pCMV G-CEPIA1er) and mitochondrial (pCMV CEPIA3mt) were a gift from Masamitsu Iono [49]. Fluorescence was recorded using a TE-2000 U Nikon epifluorescence microscope keeping the cells at 37 °C. Cells were excited with a monochomator (Cairns, UK) and the emitted fluorescence was collected every 2 s by the high sensitivity CCD EG-ORCA camera (Hamamatsu Photonics, Japan) using a 40x oil objective (Nikon, Japan). The resulting images were analyzed through MetaFluor Software (Universal Imaging, Bedford Hills, NY, USA). For experiments using fura-2/AM, the ratio between fluorescence after excitation at 340 and 380 nm was calculated, whereas for experiments using GECI the fluorescence of the CEPIA indicators at a given time point was normalized to the initial fluorescence (F/F_0_). Ratiometric fluorescence values were obtained using the MetaFluor software and selecting individual cells (ROI). Two to four coverslips with 15 to 25 cells per coverslip were analyzed for each condition. Data values were further analyzed with GraphPad Prism 6.

### Plasmid transfection and siRNA silencing

Cells were transfected with plasmids using Lipofectamine 2000 (Thermo Fisher Scientific) while transfection with siRNA to achieve silencing was accomplished with Lipofectamine RNAiMAX (Thermo Fisher Scientific). Briefly, lipofectamine and DNA or siRNA were added to medium without FBS and antibiotics. This solution was kept at room temperature for 20 min and administered to the cells. After 5 h, the medium was replaced by growth medium. The quantities of lipofectamine and DNA or siRNA were: 9 μL of lipofectamine and 2.5 μg/mL GECI plasmids; 12 μL of lipofectamine and 4.5 μg/mL of *APOE* plasmids (described in [50]), and 4.5 μL of lipofectamine and 1 μg/mL of *APOE* siRNA (s194291, Thermo Fisher Scientific) or negative control 1 μg/mL siRNA (Thermo Fisher Scientific).

### Lysosomal pH measurement

Lysosomal pH was measured using the ratiometric dye LysoSensor Yellow/Blue DND-160 (Thermo Fisher Scientific) as described [51] with minor modifications. Briefly, cells were incubated with 2 μM of LysoSensor Yellow/Blue in an isotonic solution with addition of 10% FBS. Next, cells were incubated with either additional isotonic solution for pH measurement, or with pH calibration buffers with the corresponding FBS content to perform a standard curve for each condition. 15 μM of monensin and 30 μM of nigericin were added to the pH-calibration solutions to force lysosomal pH to equilibrate with a range of pH values (solutions at 4.0, 4.5, 5.0, 5.5 and 6.0 pH). Fluorescence was measured with a GENios Pro Fluorometer, and recorded using the XFluor4GENiosPro software package (TECAN). Lysosomal pH was determined from the ratio of excitation light at 340 nm and 390 nm (collection emission at 535 nm) after extrapolation with the standard curve.

### Immunocytochemistry

Cells were fixed with 4% paraformaldehyde for 15 min and permeabilized by adding 0.1% of Triton buffer. Then, 5% of normal goat serum (NGS) was used to block the unspecific unions. Primary antibodies (mouse monoclonal anti-ApoE (sc53570, Santa Cruz Biotechnology, dilution 1:400) and rat monoclonal anti-Lamp1 (1D4B, Hybridoma Bank, dilution 1:200) were incubated overnight, followed by one-hour incubation with the secondary antibody Cy3-donkey anti-rat IgG (712–165-150, Jackson Immunoresearch, dilution 1:200) and Alexa fluor 488 goat anti-mouse IgG (A11029, Thermo Fisher Scientific, dilution 1:1000). Coverslips were mounted on a slide with Fluoromount G. Images were acquired with confocal laser scanning microscopy ZEISS LSM 700. Analysis of lysosome localization was carried out using the ImarisCell tool of IMARIS software (Bitplane), which permits manual selection of the nucleus and the membrane of each cell and computes the distance (μm) from each Lamp1-positive vesicle to the nucleus center. Lamp1-positive vesicles larger than 0.5 μm were considered. The frequency distribution of vesicles from the nucleus center to the cellular membrane was represented.

### Cholesterol staining

Filipin III (Sigma-Aldrich) was used to stain cholesterol. Cells were fixed with 4% paraformaldehyde for 15 min and incubated with 25 μg/mL Filipin for 30 min at room temperature in the dark. Images were acquired with a CCD ORCA-EG monochromatic camera (Hamamatsu) and the Eclipse TE-2000E (Nikon) epifluorescence microscope, using a 20x objective.

### Lysosomal isolation

Lysosomes from astrocytes were isolated as described [52]. Briefly, collected cells were washed in sucrose 0.25 M (pH 7.2) and disrupted in a nitrogen cavitation chamber (Kontes Glass Company), followed by homogenization in a Teflon-glass homogenizer and centrifuging (2500 xg for 15 min). The mitochondria-lysosomal-enriched faction was collected after 17,000 xg centrifugation. The lysosomal-mitochondrial-enriched fraction was then loaded in two subsequent discontinuous metrizamide/sucrose/percoll density gradients from which lysosomal pure fractions were isolated [52]. Lysosomes were broken by 5 consecutive freeze/thaw cycles and after centrifugation at 100.000 xg 30 min, lysosomal membrane and intralysosomal content were collected separately. The former pool was used for lipidomic analysis.

### Lipidomics

The extraction of metabolites for the untargeted lipidomic assay was carried out by adding 200 μL CHCl_3_:MeOH in a proportion of 1 to 2 with 0.1% formic acid to promote the ionization of molecules. Then, samples were vortexed, centrifuged at 4000 rpm, and analyzed with mass spectrometry using the QSTAR XL hybrid system (Applied Biosystems, Foster City, CA, USA). The sample was injected at a flux of 15 μL/min through the infusion integrated pump. Spectra were acquired during positive ionization in a range of m/z from 50 to 1100 uma. The ionization parameters were: 3300 V of voltage ion spray, 60 V of decluttering potential, and 250 V of focusing potential. Nitrogen was used as a collision gas for the spectra acquisition. Markerview™ and SIMCA-P™ software were used to reduce the results into a two-dimensional matrix of peak spectra and intensity of peaks, and for the statistical analysis. Inter-genotype comparisons were carried out with the multivariate analysis Partial Least Squares-discriminant analysis (PLS-DA). Next, the Variable Importance in Projection (VIP) was used to establish which metabolites had more impact in the segregation of samples according to *APOE3* and *APOE4* genotypes. ANOVA with a Tukey correction post-test was applied to the group of metabolites with a VIP > 1 to assess, again, inter-genotype differences with the identified metabolites. We then proceeded to identify metabolites with VIP > 1 comparing their accurate masses with those available in metabolomics databases (HMDB, METLIN, KEGG and LIPIDMAPS) [53]. Finally, fold changes of identified metabolites in *APOE4* vs *APOE3* astrocytes were calculated and a multi-t statistical test with corrected probability (False Discovery Rate –FDR- 5%) was applied.

### Western blot

Cells for protein extraction were lysed with RIPA buffer and extracts were sonicated and centrifuged. Protein extracts were quantified with BCA kit (23,225, Thermo Fisher Scientific) according to the manufacturer’s protocol. 20 μg protein of samples was loaded in 12% polyacrylamide gels electrophoresis (PAGE). Electrophoresis was conducted at a constant amperage (30 mA) for approximately 2 h followed by the protein transference to a PVDF membrane at constant voltage (100 mV) for 1.5 h. 5% non-fat milk was used to block unspecific unions, and the primary antibodies mouse monoclonal anti-V-ATPase subunit V_0_D_1_ (ab56441, Abcam, used at 2.5 μg/mL) and monoclonal anti β-actin (a5316, SigmaAldrich, used at 1/20000), were incubated overnight. The next day, the secondary antibody was incubated for 1 h (goat anti-mouse IgG: 31430, Thermo Fisher Scientific, used at 1/10000). Finally, the membrane was developed using the chemiluminescence kit of BioRad according to the manufacturer’s protocol, and membrane chemiluminescence was detected with a Chemidoc MP Image System (BioRad). Image lab software (BioRad) was used for the quantification of bands.

### Measurement of mRNA expression

Cells were collected adding Trizol Reagent. Then, 0.2 mL chloroform was added to the extracts, and these were centrifuged at 11,500 rpm for 15 min at 4 °C allowing the formation of three phases. The RNA-containing phase (superior phase) was isolated by pipetting carefully, and 0.2 mL of isopropanol was added to precipitate of RNA, which was then washed with cold ethanol 75%. RNA-sample concentration was determined with a Nanodrop 200 spectrophotometer (Thermo Fisher Scientific). 2 μg of RNA was reverse transcripted to cDNA using 1 μM of oligo DT, 1 μM of hexamers, 0.5 mM dNTPs, 0.45 mM DTT, 10 U RNAse out, RT buffer, and 200 U of retrotranscriptase. The PCR program was divided into four steps: 65 °C for 10 min, 25 °C for 10 min, 42 °C for 1 h, and 72 °C for 10 min.

Gene expression was carried out with quantitative real-time PCR (qPCR) using Taqman and SYBR green technology. Fluorescence was detected with the 7500 Fast Real-Time PCR System. qPCR cycles were the following: a holding stage of 50 °C (2 min), 10 min at 95 °C and 40 cycles of 95 °C (15 s), and 60 °C (1 min). A similar protocol was used for SYBR assay but with an extra stage for the melting curve (15 s at 95 °C, 1 min at 60 °C, 30 s at 95 °C, and 15 s at 60 °C). Data analysis was performed using Cq value, and the average of the gene efficiency provided by LinReg PCR software, following the formula 1 + eficience^ΛCq^ of each gene analyzed. Expression data were normalized with housekeeping genes (Gapdh and/or18s), using their geometric mean calculated according to the geNorm algorithm [54]. TaqMan primers of *APOE* (Hs00171168_m1), Trpml (Mm00522550_m1), Gapdh (Mm9999915_g1), and 18S (Mm03928990) were purchased from Thermo Fisher Scientific. Primers for SYBR green were designed according to the sequence of the gene: Tpc1 (5′-CTGGGAGAGATGAATTATCAAGAG-3′; 5′-GTTGTGTACGAAGAGGTAGG-3′), Tpc2 (5’GCTGAGCCTTGCTTGTGAGG-3′; 5′-ACACTTCAGGGTCTTCTTCATCA-3′), and Gapdh (5′-AAGCTCATTTCCTGGTATGAC-3′; 5′-TGGTCCAGGGT TTCTTACTC-3′).

### Statistical analysis

Each determination was carried out with cells from at least three different passages. A parametric unpaired T-test was used for the comparison of a given variable in two different conditions or cell types, whereas one-way or two-way analysis of variance (ANOVA), with a Tukey’s or Dunn’s post hoc test, was used when comparing more than two conditions. The software employed was GraphPad Prism 6, and data are represented as mean ± SEM (standard error of the mean). A *p*-value < 0.05 was considered significant (*p*-value < 0.05 (*), *p*-value < 0.01 (**), *p*-value < 0.001 (***) and *p*-value < 0.0001(****).

## Results

### *APOE4* expression alters astrocytic excitability

**Fig. 1:**
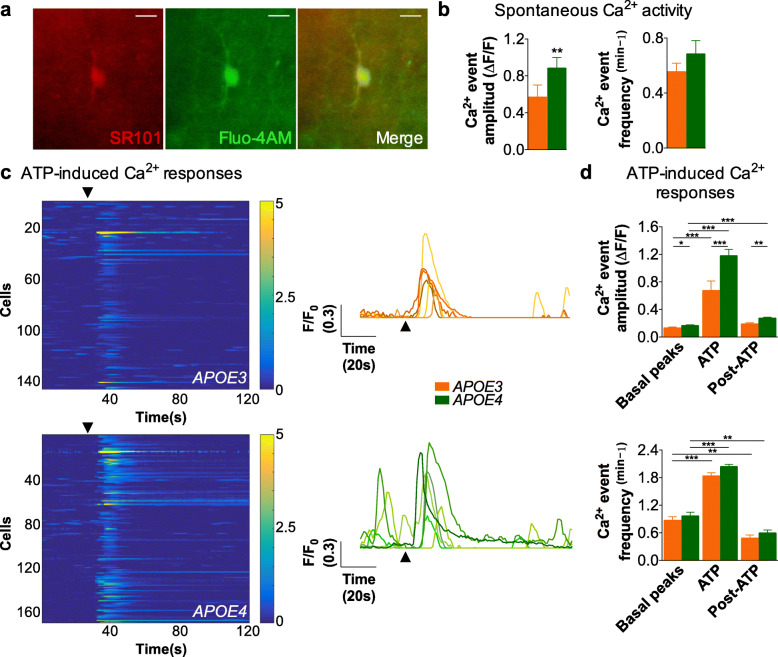
Enhanced Ca^2+^ signals in astrocytes from male *APOE4* vs *APOE3* targeted replacement mice. **a** Astrocyte from *stratum radiatum* of hippocampus of male mice labeled with SR101 (red), fluo-4/AM (green), and merged image. Scale bar represents 10 μm. **b** Spontaneous Ca^2+^ activity in astrocytes from *APOE3* (*N* = 36 astrocytes) and *APOE4* male mice (*N* = 16 astrocytes). Ca^2+^ was monitored for 120 s without any stimulation. **c** Left panels, raster plots of Ca^2+^ activity in *APOE3* (upper panel, *N* = 146 astrocytes), and *APOE4* male mice (lower panel, *N* = 171 astrocytes). The color code indicates relative fluorescence changes before and after local application of ATP (arrow, 5 s, 1 mM). Right panel, representative traces of Ca^2+^ signals evoked by an ATP puff in *APOE3* (top) and *APOE4* (bottom) astrocytes (arrows indicate ATP stimulation). **d** Quantification of the amplitude and frequency of Ca^2+^ events for 30 s before (basal peaks), during (ATP) and after local application of stimulus (post-ATP). Statistical significance was established at *p* < 0.05 (*), *p* < 0.01 (**), and *p* < 0.001 (***); One-way ANOVA followed by Dunn’s post hoc test. All the experiments were performed in the presence of TTX (1 μM). *N* = 4 mice, for both *APOE3* and *APOE4*

To determine whether expression of the allele *APOE4* alters astrocyte excitability, we recorded Ca^2+^ in hippocampal slices of 9–12-week-old male and female mice in which the endogenous mouse *APOE* gene had been replaced with human *APOE3* or *APOE4* genes. Recordings were made in ACSF medium supplemented with 10% FBS, in order to keep lipoprotein and lipid concentration as close as possible to physiological conditions. Slices were incubated with the Ca^2+^ indicator fluo-4/AM and the astrocytic marker sulforhodamine (SR101) (Fig. 1a, see Materials and methods). We analyzed Ca^2+^ spontaneous activity—that is, Ca^2+^ events at rest conditions—and neurotransmitter-induced Ca^2+^ responses in cells co-labeled with Fluo-4/AM and SR101. To study spontaneous activity, we recorded *APOE3* and *APOE4* astrocytes for 120 s. For receptor-mediated Ca^2+^ responses, we recorded basal peak activity for 30 s, and then we stimulated slices with 1 mM ATP. We selected purinergic stimulation because it elicits Ca^2+^ signals both in astrocytes in vivo and in vitro, triggering several physiological functions [34, 55], and because responses elicited by stimulation of purinergic receptors are the cause of Ca^2+^ hyperactivity in AD mouse models [56].

In male mice, we detected increased amplitude of spontaneous Ca^2+^ transients in *APOE4* vs *APOE3* astrocytes (Fig. 1b). Regarding induced activities, purinergic stimulation caused in both genotypes the increase in magnitude and frequency of Ca^2+^ transients typically seen in astrocytes. The amplitude of Ca^2+^ responses was greater in *APOE4* cells than in *APOE3* cells (Fig. 1c and d). Further, the magnitude of Ca^2+^ responses decreased after removal of ATP (post ATP phase) to basal levels in *APOE3* astrocytes, but remained significantly increased over its own basal levels, and with respect to *APOE3* cells, in *APOE4* cells (Fig. 1c and d). The frequency of Ca^2+^ responses was similar in both genotypes, in both the ATP and post-ATP phases. It is worth stressing that the amplitude of spontaneous Ca^2+^ transients was also statistically increased in *APOE4* compared to *APOE3* cells in these set of experiments, confirming the results of the 120-s recordings (Fig. 1c and d).

**Fig. 2:**
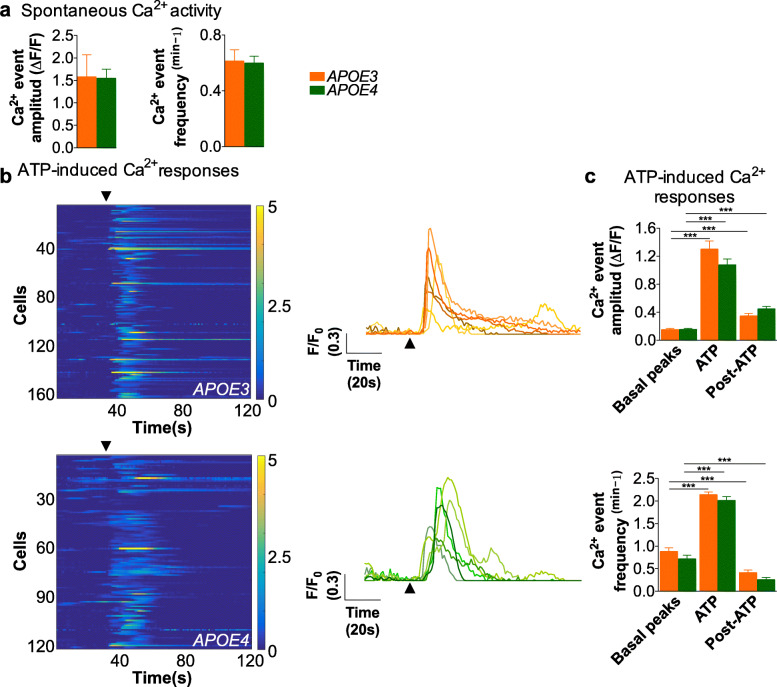
Equal Ca^2+^ signals in astrocytes from female *APOE3* and *APOE4* targeted replacement mice. **a** Spontaneous Ca^2+^ activity in astrocytes from *APOE3* (*N* = 26 astrocytes) and *APOE4* female mice (*N* = 35 astrocytes). Ca^2+^ was monitored for 120 s without any stimulation. **b** Left panels, raster plots of Ca^2+^ activity in astrocytes from *APOE3* (upper panel, *N* = 165 astrocytes), and *APOE4* female mice (lower panel, *N* = 124 astrocytes). The color code indicates relative fluorescence changes before and after local application of ATP (arrow, 5 s, 1 mM). Right panel, representative traces of Ca^2+^ signals evoked by an ATP puff in *APOE3* (upper panel) and *APOE4* (bottom panel) astrocytes (arrows indicate ATP stimulation). **c** Quantification of the amplitude and frequency of Ca^2+^ events for 30 s before (basal peaks), during (ATP) and after local stimulus (post-ATP) in *APOE3* and *APOE4* astrocytes. Statistical significance was established at *p* < 0.001 (***); One-way ANOVA followed by Dunn’s post hoc test. All the experiments were performed in the presence of TTX (1 μM). *N* = 4 mice, for both *APOE3* and *APOE4*

Female mice differed from males in two respects. First, the amplitude of spontaneous and ATP-induced events was significantly increased (*p* < 0.001), by at least 2-fold, in astrocytes from *APOE3* females as compared to *APOE3* males (compare Fig. 1b with 2a and 1d with 2c). Second, no differences were observed between *APOE3* and *APOE4* astrocytes in females in the magnitude of spontaneous and induced events (Fig. 2a-c). This may suggest that Ca^2+^ responses in astrocytes from *APOE3* females represented the maximal Ca^2+^ response that could not increase further. Altogether, the ex vivo observations suggest that expression of human *APOE* alleles modulates Ca^2+^ transients in astrocytes in a sex-specific manner, such that expression of the *APOE3* allele in male mice results in globally decreased Ca^2+^ transients in astrocytes—or expression of *APOE3* in females in a global increase—as compared to the *APOE4* allele.

### Immortalized *APOE4* astrocytes show increased Ca^2+^ mobilization from acidic stores

**Fig. 3:**
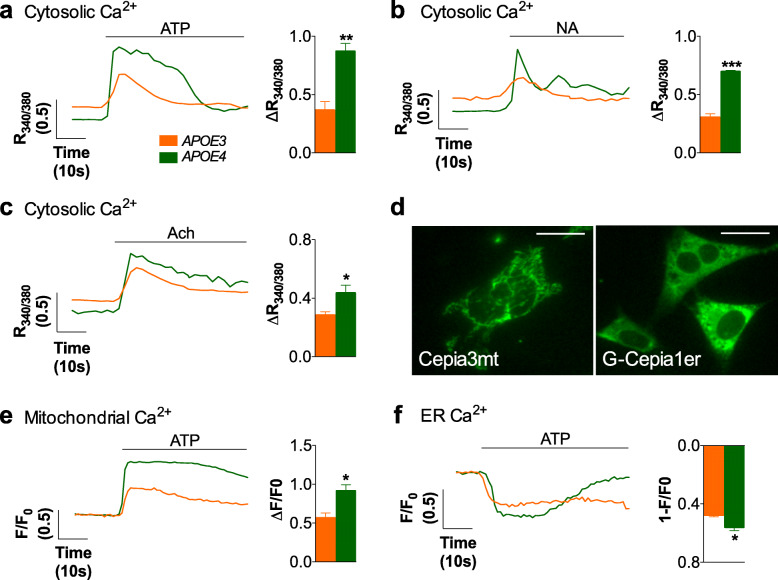
Enhanced Ca^2+^ signals in immortalized astrocytes from *APOE4* vs *APOE3* targeted replacement mice. Ca^2+^ responses measured using fura-2/AM in *APOE3* and *APOE4* astrocytes after stimulation with (**a)** 100 μM ATP, (**b)** 10 μM noradrenaline (NA), and (**c**) 100 μM acetylcholine (Ach). Representative traces and quantification of the magnitude of the responses are shown (*N* = 4 for **a** and **c**, and *N* = 3 for **b**). **d** Images of astrocytes transfected with the Ca^2+^ probes for mitochondria (CEPIA3mt) and ER (G-CEPIA1er). Scale bar represents 15 μm. **e** Representative traces and quantification of mitochondrial Ca^2+^ in *APOE3* and *APOE4* cells transfected with CEPIA3mt upon stimulation with ATP (*N* = 3). **f** Representative traces and quantification of the decrease in ER Ca^2+^ upon stimulation of purinergic receptors in *APOE3* and *APOE4* cells transfected with GCEPIA1er (*N* = 5). Unpaired parametric T-test was used to compare responses in *APOE3* vs *APOE4* astrocytes. *p* < 0.05 (*), *p* < 0.01 (**), *p* < 0.001 (***)

To gain insight into the mechanism by which expression of different *APOE* alleles regulates Ca^2+^ transients in astrocytes, we used immortalized astrocytes that express human *APOE3* or *APOE4* [44] since this in vitro model allows for experimental manipulations that are not feasible in brain slices. As with slices, cells were supplemented with 10% FBS, in order to keep lipoprotein and lipid concentration as close as possible to physiological conditions. It is worth stressing that immortalized astrocytes are aneuploid so the sexual identity is lost. Thus, a key question was whether their Ca^2+^ phenotype is male- or female-like. Our data show that they reproduce a male-like Ca^2+^ signaling phenotype in the presence of lipids. First, differences in Ca^2+^ responses at rest showed the same trend observed in male mice. *APOE4* astrocytes had significantly different Ca^2+^ basal levels than *APOE3* astrocytes (*p*-value = 0.01), the fluorescence ratio being 0.28 ± 0.01 and 0.41 ± 0.03, respectively (Fig. 3a-c). Note that cultured astrocytes do not show at rest the so-called spontaneous Ca^2+^ oscillations observed ex vivo, but stable basal Ca^2+^ levels that we could compare thanks to the ratiometric fura-2/AM Ca^2+^ indicator. Second, 100 μM ATP stimulation resulted in greater Ca^2+^ responses in *APOE4* than in *APOE3* astrocytes (Fig. 3a). Purinergic-induced Ca^2+^ responses also lasted longer: the response was 64.9 ± 8.2% of the maximum peak signal after 20 s in *APOE4* cells but only 27.7 ± 1.5% in *APOE3* astrocytes. Moreover, altered Ca^2+^ signaling was not restricted to purinergic stimulation, as adrenergic and muscarinic-receptor activation also triggered greater cytosolic Ca^2+^ responses in *APOE4* than in *APOE3* astrocytes (Fig. 3b and c). Importantly, the magnitude of purinergic-induced Ca^2+^ responses was the same with two other FBS batches (peak responses after stimulation with 100 μM ATP were 0.36 ± 0.13 in *APOE3* and 0.94 ± 0.01 in *APOE4*; and 0.37 ± 0.04 in *APOE3* and 0.91 ± 0.05 in *APOE4* astrocytes). Taken together, the data support immortalized *APOE3* and *APOE4* astrocytes as a model to study the mechanisms underlying the regulation of Ca^2+^ responses by *APOE* alleles in males.

**Fig. 4:**
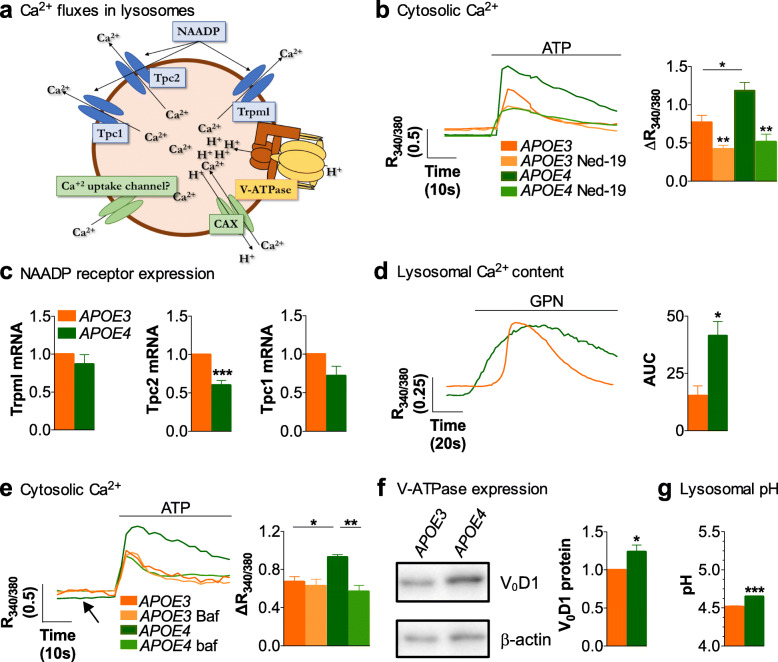
Dysregulated Ca^2+^ excitability in *APOE4* immortalized astrocytes is due to greater lysosomal V-ATPase activity. **a** Schematic of the lysosomal Ca^2+^ mobilization and uptake mechanisms. **b** Representative traces and quantification of ATP-induced cytosolic Ca^2+^ responses of cells treated with 0.1% DMSO or 100 μM Ned-19 (diluted in 0.1% DMSO) for 20 min (*N* = 3). **c** Quantification by real time qPCR of mRNA expression of Tpc1 and Tpc2 (normalized to Gapdh mRNA contents, *N* = 5) and Trpml (normalized to Gapdh mRNA contents, *N* = 4). **d** Representative traces and quantification of the area under the curve (AUC) of intracellular Ca^2+^ after lysing the lysosomes with 200 μM GPN (*N* = 4). **e** Representative traces and quantification of ATP-induced Ca^2+^ responses after treating cells with DMSO or 2 μM bafilomycin A1 (Baf) for 20 min (*N* = 3). Note that the black arrow indicates differences in basal Ca^2+^. **f** Quantification of V-ATPase subunit V_0_D_1_ with western blot, normalized to β-actin expression (*N* = 4). **g** Quantification of the pH of acidic organelles in *APOE3* and *APOE4* astrocytes (*N* = 3). Unpaired parametric T-test was used, except in **b** and **e**, where one-way ANOVA was used. *p* < 0.05 (*), *p* < 0.01 (**), *p* < 0.001 (***)

Next, in order to identify which pathways are dysregulated in *APOE4* astrocytes, we examined Ca^2+^ fluxes among the principal intracellular Ca^2+^ sources with organelle-specific probes and pharmacological manipulations. First, we investigated the mitochondrial Ca^2+^ uptake that characteristically buffers increases in cytosolic Ca^2+^. Since *APOE4* has been described as harming mitochondria in neurons [57], we reasoned that harmed mitochondria in *APOE4* astrocytes could result in deficient Ca^2+^ uptake, and hence in increased intracellular Ca^2+^ responses. However, expression of the mitochondrial Ca^2+^ indicator CEPIA3mt (Fig. 3d) showed higher Ca^2+^ uptake in *APOE4* mitochondria compared to *APOE3* astrocytes, consistent with the higher ATP-induced Ca^2+^ responses in the cytosol (Fig. 3e). Second, we studied the main Ca^2+^ signaling pathway in astrocytes, Ca^2+^ mobilization from the ER through the IP3 receptor, by transfecting cells with G-CEPIA1er (Fig. 3d), and directly measuring Ca^2+^ contents inside this organelle. As expected, 100 μM ATP decreased Ca^2+^ levels in the ER of both *APOE3* and *APOE4* astrocytes, indicative of Ca^2+^ being released to the cytosol. Since the process is, although significantly, just slightly reinforced in *APOE4* astrocytes (Fig. 3f), the greater cytosolic Ca^2+^ responses in these cells could not rely exclusively on increased Ca^2+^ mobilization from the ER. Third, we explored Ca^2+^ mobilization from acidic stores, which are mainly lysosomes and related organelles [58] that we have shown to be involved in purinergic-induced Ca^2+^ responses in astrocytes [37]. Figure 4a shows the main Ca^2+^ fluxes in lysosomes. We recorded cytosolic Ca^2+^ with fura-2/AM, after inhibiting Ca^2+^ release from acidic stores with 100 μM of Ned-19, an inhibitor of NAADP receptors responsible for Ca^2+^ release from these organelles [59]. Control cells were treated with DMSO, the vehicle of Ned-19. As expected, Ned-19 reduced ATP-induced Ca^2+^ responses in *APOE3* cells (Fig. 4b), confirming the contribution of lysosomal Ca^2+^ to cytosolic transients [37]. Interestingly, Ned-19 greatly reduced Ca^2+^ responses in *APOE4* astrocytes, such that purinergic-mediated Ca^2+^ responses in the presence of Ned-19 were of similar magnitude in both cell types. Hence, Ca^2+^ release from acidic-stores appears to be responsible for the greater purinergic-elicited Ca^2+^ responses in *APOE4* astrocytes. Altogether, our results thus far lend support to the idea that lysosomal-related Ca^2+^ release is the main Ca^2+^ signaling pathway dysregulated in *APOE4* astrocytes.

### Dysregulation of V-ATPase activity contributes to the alteration of Ca^2+^ responses in *APOE4* astrocytes

Ca^2+^ is released from acidic stores upon stimulation of NAADP receptors, which are Ca^2+^ channels, the most accepted candidates being two-pore channels 1 and 2 (Tpc1, Tpc2), and transient receptor potential mucolipin (Trpml) [59]. By contrast, Ca^2+^ uptake by acidic stores is accomplished through an indirect mechanism whereby V-ATPase pumps H^+^ into the vesicles, and then H^+^ are exchanged with Ca^2+^ through the Ca^2+^/H^+^ exchanger (CAX) [60] (Fig. 4a). Thus, greater Ca^2+^ release from acidic stores in *APOE4* astrocytes might be due to two phenomena: greater expression or activation of NAADP receptors, or greater Ca^2+^ stored in these organelles, due to increased activity of V-ATPase and/or CAX. First, we analyzed the expression of NAADP receptors in astrocytes with real time qPCR. Expression of Trpml and Tpc1 channels is the same in *APOE3* and *APOE4* cells, while expression of Tpc2 channel is lower in *APOE4* cells (Fig. 4c), perhaps as a result of a negative feedback mechanism in response to the high Ca^2+^ signals in *APOE4* astrocytes. In any event, the increased release of Ca^2+^ from *APOE4* lysosomes cannot be attributed to increased expression of NAADP receptors.

Second, we analyzed Ca^2+^ contents inside acidic stores with a protocol in which they were osmotically lysed due to the accumulation of the peptide Glycyl-L-phenylalanine 2-naphthylamide (GPN), followed by its proteolysis by cathepsin C, such that the stored lysosomal Ca^2+^ is released into the cytoplasm. Then, the area under the curve (AUC) of the cytosolic Ca^2+^ increase after addition of GPN to the astrocytes was used to calculate the total amount of stored calcium in these organelles. The results showed greater AUC in *APOE4* cells (Fig. 4d), supporting a higher concentration of lysosomal-related Ca^2+^ in *APOE4* cells, as compared to *APOE3*. Since there are no pharmacological modulators of CAX, we relied on a pharmacological inhibitor of V-ATPase, bafilomycin A1, and on measurements of pH in acidic organelles, to establish the implication of the pump and CAX in the increased luminal Ca^2+^. Bafilomycin A1 increased basal cytoplasmic Ca^2+^ concentration in *APOE4* astrocytes to 0.40 ± 0.05 (control values of astrocytes treated with vehicle were 0.30 ± 0.01; *p*-value = 0.05), whereas there was no effect on non-stimulated intracellular Ca^2+^ levels in *APOE3* (0.44 ± 0.08 for cells treated with bafilomycin A1 compared to 0.39 ± 0.02 of cells treated with vehicle). This suggests that the aforementioned lower basal cytoplasmic Ca^2+^ concentration in *APOE4* cells is due to greater V-ATPase-mediated Ca^2+^ uptake into acidic stores, consistent with the greater intralysosomal Ca^2+^ levels revealed by the GPN experiments. Accordingly, bafilomycin A1 reduced the ATP-induced Ca^2+^ release in *APOE4* astrocytes, confirming the dependence of these Ca^2+^ responses on acidic stores (Fig. 4e). In contrast, bafilomycin A1 did not change purinergic-induced Ca^2+^ responses in *APOE3* astrocytes. Thus, both basal levels and ATP responses in the presence of bafilomycin A1 might indicate the existence of low V-ATPase activity in *APOE3* astrocytes. Indeed, the expression of V-ATPase subunit V_0_D_1_, which is highly expressed in astrocytes [61], was higher in *APOE4* cells than in *APOE3* cells (Fig. 4f). Because V-ATPase controls the flow of H^+^ into acidic stores, we analyzed the pH of acidic stores with the probe lysosensor and fluorometry. We found that both *APOE3* and *APOE4* astrocytes had standard lysosomal-related pH, with a minimal but significant difference of 0.14 pH units between genotypes, *APOE4* lysosomes being more alkaline (Fig. 4g). If the pH does not decrease despite increased activity of the V-ATPase pump, it follows that CAX activity must be increased in *APOE4* astrocytes, too, such that the increased number of H^+^ entering the acidic stores exit through CAX in exchange for Ca^2+^. As a result, lysosomal Ca^2+^ concentration and hence basal Ca^2+^ and purinergic-induced Ca^2+^ release from lysosomes, are greater in *APOE4* than in *APOE3* astrocytes. Note that we did not determine nor manipulate CAX expression in *APOE3* and *APOE4* cells because the sequence of mouse CAX is unknown.

### *APOE3* expression in *APOE4* astrocytes reduces cytosolic Ca^2+^ responses

**Fig. 5:**
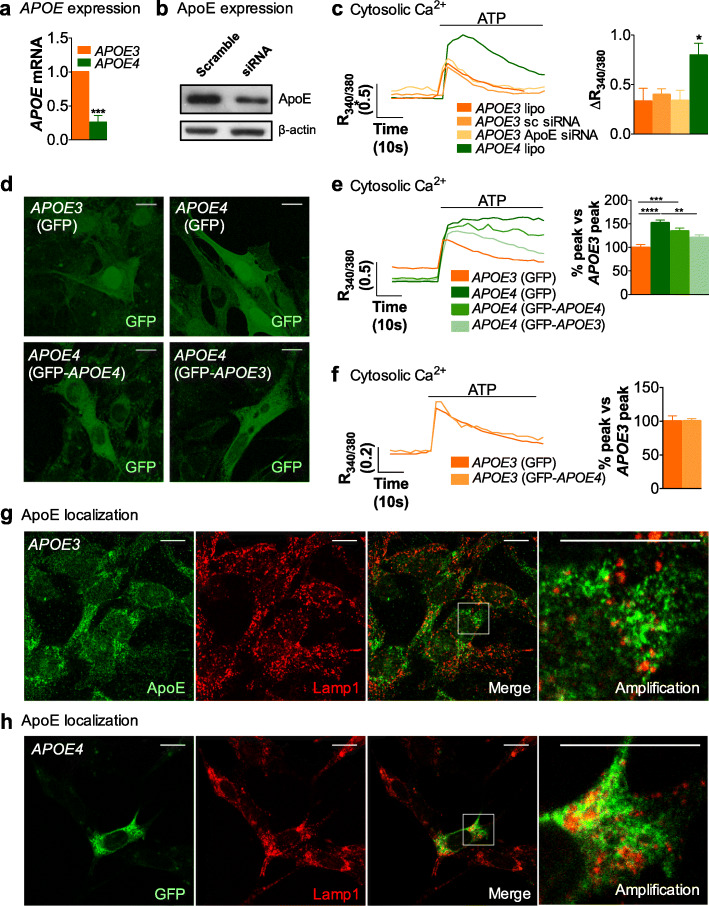
Effects of the *APOE* allele vs APOE contents on Ca^2+^-based excitability. **a***APOE* mRNA expression relative to Gapdh and 18S mRNA contents in *APOE3* and *APOE4* immortalized astrocytes, quantified by real time qPCR (*N* = 3). **b** Representative ApoE western blot of *APOE3* cells transfected with scramble or *APOE* siRNA. **c** Representative traces and quantification of the magnitude of ATP-induced Ca^2+^ responses in *APOE3* astrocytes transfected with lipofectamine (lipo), plus scramble (sc) or *APOE* siRNA, and *APOE4* astrocytes treated only with lipofectamine (*N* = 4). **d** Representative images of immunocytochemistry of *APOE3* and *APOE4* cells transfected with plasmids expressing GFP, GFP-*APOE4*, and GFP-*APOE3*, as indicated. Scale bar represents 15 μm. **e** Representative traces and quantification of the magnitude of 100 μM ATP-elicited Ca^2+^ responses in *APOE3* and *APOE4* cells transfected with different plasmids as indicated. Ca^2+^ peaks after ATP stimulation are relative to the responses of *APOE3* cells transfected with GFP. Only GFP fluorescent cells were analyzed (at least 30 cells from 4 independent experiments). **f** Quantification of the magnitude of Ca^2+^ responses elicited by 100 μM ATP in *APOE3* astrocytes transfected with GFP or GFP-*APOE4* plasmids, as indicated. Ca^2+^ peaks after ATP stimulation are relative to the responses of *APOE3* cells transfected with GFP. Only GFP fluorescent cells were analyzed (at least 30 cells from 4 independent experiments). **g** Representative images of Lamp1 (red) and ApoE (green) immunocytochemistry in *APOE3* cells. Merged images and amplifications of the area in the white frame, are displayed. Scale bar represents 15 μm. **h** Representative images of Lamp1 immunocytochemistry (red) and GFP-ApoE fluorescence (green) in *APOE4* cells. Merged images and the amplification of a cell of each image, indicated with a white frame, are displayed. Scale bar represents 15 μm. Unpaired parametric T-test was used in a, and one-way ANOVA in c and e. *p* < 0.05 (*), *p* < 0.01 (**), *p* < 0.001 (***), *p* < 0.0001 (****)

Is the effect of *APOE4* due to: (1) loss-of-function due to decreased contents [62], (2) malfunction, (3) gain-of-toxic function because of its structure, or (4) misplaced intracellular localization? We first examined ApoE levels in immortalized *APOE3* and *APOE4* astrocytes by quantifying mRNA levels with qPCR. As shown in Fig. 5a, *APOE* expression is lower in *APOE4* than in *APOE3* astrocytes. Therefore, it is plausible that lower expression of *APOE4* accounts for Ca^2+^ signaling alterations. To test this possibility, we modulated the quantity of *APOE3* and *APOE4* with the rationale that, if *APOE* expression matters, decreasing ApoE in *APOE3* cells would increase Ca^2+^ responses, whereas increasing ApoE in *APOE4* cells would decrease Ca^2+^ responses. Upon decreasing *APOE* expression in *APOE3* astrocytes with an *APOE* siRNA (Fig. 5b), ATP-induced Ca^2+^ responses were still lower, as compared to *APOE4* astrocytes, with no differences observed between scramble- and siRNA-transfected *APOE3* cells (Fig. 5c). We also tried the opposite strategy: we increased *APOE* expression using GFP-*APOE3* or GFP-*APOE4* plasmids, which overexpress a GFP-ApoE fusion protein, which then allows one to identify cells with increased *APOE* expression (Fig. 5d). The GFP signal did not interfere with fura-2/AM (data not shown). Because transfection efficiencies were different among experiments (5 to 50% of GFP transfected cells), we avoided inter-experimental variability by normalizing peak Ca^2+^ responses to levels recorded in *APOE3* astrocytes transfected with a GFP-expressing plasmid without *APOE* constructs. The over-expression of GFP-*APOE3*, but not of GFP-*APOE4*, transformed *APOE4* into *APOE3* astrocytes, in a statistically significant manner (Fig. 5e), suggesting that Ca^2+^ hyperactivity in *APOE4* astrocytes is mainly due to the expression of this particular isoform, but not to lower *APOE* expression. The data do not support, however, a gain of a toxic function by *APOE4*, because, if this were the case, *APOE4*-associated dysfunction would have been potentiated by GFP-*APOE4* over-expression, and would not have been rescued by GFP-*APOE3*, since the toxic element, *APOE4*, remained. In agreement, there were no differences between ATP-induced Ca^2+^ signals in *APOE3* cells transfected with GFP-*APOE4* or with GFP (Fig. 5f), again ruling out a toxic effect of *APOE4*. Taken together, our data support *APOE4* malfunction rather than toxicity.

Finally, because acidic stores accounted for the differences in Ca^2+^ responses between genotypes, we studied whether ApoE was localized in the acidic stores. Because the quality of staining of such organelles with the fluorophore lysotracker was not optimal for confocal studies (data not shown), we resorted to using Lamp1, a marker for lysosomes, autophagosomes, and different vesicles of the endolysosomal pathway, including late-endosomes [63]. In *APOE3* astrocytes, we used ApoE and Lamp-1 immunostaining (Fig. 5g). In *APOE4* astrocytes, we relied on *APOE4* overexpression with GFP-*APOE4*, since the low amounts of *APOE* expression in *APOE4* cells precluded immunocytochemical analysis (Fig. 5h). We observed that neither ApoE3 nor ApoE4 from GFP-*APOE4* colocalized with Lamp-1. These observations rule out the likelihood that the alterations in lysosomal-related Ca^2+^ handling in *APOE4* astrocytes are due to loss of a direct interaction of ApoE with channels mediating Ca^2+^ fluxes, suggesting, instead, indirect actions of the apolipoprotein, perhaps through changes in lipid homeostasis.

### ATP-induced Ca^2+^ responses are modulated by extracellular lipids in *APOE3* but not *APOE4* astrocytes

Considering the wealth of evidence documenting how channels and pumps involved in Ca^2+^ signaling are regulated by lipids, particularly by membrane lipids [64, 65], we posited that lipids regulate Ca^2+^ fluxes in astrocytes, too, and that aberrant lipid homeostasis accounts for the observed dysregulation of Ca^2+^ fluxes in *APOE4* astrocytes, particularly in lysosome-related organelles. Lipid modulation of Ca^2+^ transients in astrocytes is uncharted territory. Thus, to gain insight into the control of astrocyte excitability by lipids in the context of *APOE4*, we carried out two sets of experiments. First, we aimed to obtain proof-of-concept that lipids regulate Ca^2+^ transients in astrocytes, by examining the effect of changing lipid contents on the excitability of immortalized *APOE3* and *APOE4* astrocytes. Second, we analyzed cholesterol subcellular distribution, and we performed a lipidomic analysis of lysosomal and whole-membrane to identify candidates for excitability-modulating lipids, and changes thereof in the two genotypes.

**Fig. 6:**
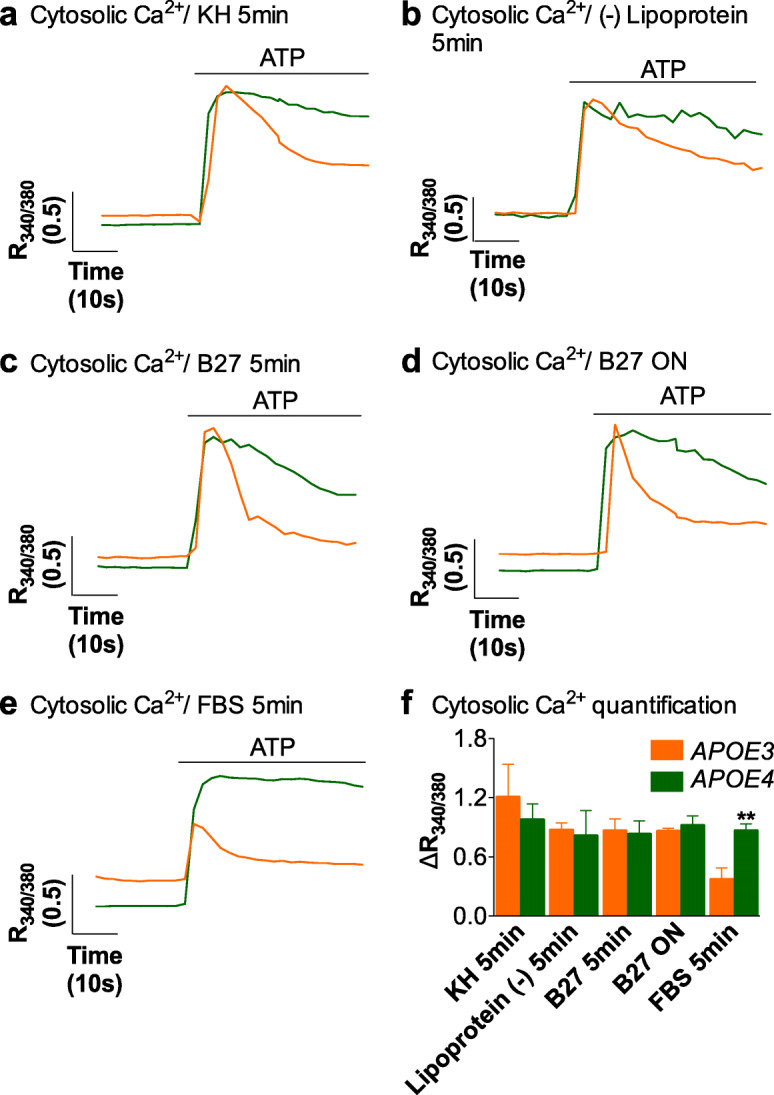
Loss of modulation of Ca^2+^ signals by lipids in *APOE4* astrocytes. Representative traces of 100 μM ATP-induced Ca^2+^ responses in *APOE3* and *APOE4* immortalized cells in (**a**) saline medium or Krebs medium (KH) for 2 to 5 min, (**b**) DMEM medium supplemented with lipoprotein-deficient serum (Lipoprotein (−)) for 2 to 5 min, (**c**) DMEM medium supplemented with B27 for 2 to 5 min, and (**d**) overnight (ON), and (**e**) DMEM medium supplemented with 10% FBS for 5 min. **f** Quantification of the ATP-induced intracellular Ca^2+^ peak in cells kept in the different mediums (*N* = 3). Two-way ANOVA was used. *p* < 0.01 (**) as compared to *APOE3* cells kept in the same medium

Thus far, all our Ca^2+^ imaging experiments had been performed in medium supplemented with FBS, rich in nutrients, including lipoproteins. Here, we replaced this medium with three different media with lower lipid composition 2–5 min prior to Ca^2+^ imaging: 1) Krebs medium (KH), 2) DMEM supplemented with lipoproteins-deficient FBS and 3) DMEM with B27, a supplement without lipoproteins and restricted composition of lipids: linoleic acid, linolenic acid, progesterone, and corticosteroids. In the absence of lipids, non-stimulated basal Ca^2+^ levels were significantly lower in immortalized *APOE4* vs *APOE3* astrocytes, as detected in the presence of lipids. Thus, in KH, basal levels were 0.29 ± 0.02 in *APOE3* vs 0.23 ± 0.03 in *APOE4* (*p*-value = 0.04); in DMEM supplemented with lipoprotein-deficient FBS, 0.39 ± 0.07 in *APOE3* vs 0.29 ± 0.06 in *APOE4* (p-value = 0.04); and in DMEM with B27, 0.30 ± 0.02 in *APOE3* vs 0.22 ± 0.01 in *APOE4* (p-value = 0.007). By contrast, in all three conditions, stimulation of purinergic responses elicited Ca^2+^ responses of similar magnitude in *APOE3* and *APOE4* astrocytes (Fig. 6a-c, f). It was not the case that responses in *APOE4* cells had diminished, but, rather, that responses had increased in *APOE3* cells. That is, *APOE4* astrocytes present the same magnitude of ATP-induced Ca^2+^ signals regardless of the lipids presents, whereas *APOE3* astrocytes adapt their Ca^2+^ responses to the concentration of extracellular lipids, with responses being low in media rich in lipids (presence of FBS) and high in the presence of low lipid concentration, or no lipids. Consistent with the greater ATP-triggered Ca^2+^ responses, the responses also lasted longer in *APOE3* astrocytes in the absence of lipids: the response was 36.0 ± 4.0% of the maximum peak signal after 20 s in KH, 51.6 ± 4.3% in DMEM supplemented with lipoproteins-deficient FBS and 34.6 ± 3.0% in DMEM with B27, as compared to the previously reported 27.7 ± 1.5% in DMEM with FBS (*p* < 0.05). Thus, the results point to a potentiation of long-lasting amplification Ca^2+^ pathways, perhaps by Ca^2+^-induced Ca^2+^ release from the ER or store-operated Ca^2+^ entry (SOCE)—that is, Ca^2+^ influx across the plasma membrane in response to depletion of intracellular Ca^2+^ stores. Still, ATP responses were significantly shorter (*p*-value < 0.01) in lipid-depleted *APOE3* astrocytes as compared to *APOE4* astrocytes (72.57 ± 3%, 68.4 ± 6.2% and 81.2 ± 6.8% for *APOE4* cells kept in KH, DMEM with lipoprotein-deficient FBS and DMEM with B27), indicating that some differences in Ca^2+^ fluxes remain between *APOE3* and *APOE4* astrocytes.

It is worth stressing that the increased ATP-induced Ca^2+^ response in *APOE3* astrocytes is a fast-onset process, as it was observed just a few min after lipoprotein removal. It is not transient, because it persisted 12 h after replacement of DMEM supplemented with FBS for DMEM supplemented with B27 (Fig. 6d and f), and it is reversible, because it diminishes if the medium is replaced again by DMEM supplemented with FBS 5 min prior to Ca^2+^ recordings (Fig. 6e and f).

Altogether, three conclusions may be drawn from these results: they constitute the first demonstration that astrocyte excitability is modulated by lipids and/or lipoproteins, such regulation is reversible but stable in *APOE3* astrocytes as long as lipids remain present, and is lost in *APOE4* astrocytes.

### The absence of lipids potentiates Ca^2+^ release from the ER and extracellular Ca^2+^ entry in *APOE3* but not *APOE4* astrocytes

**Fig. 7:**
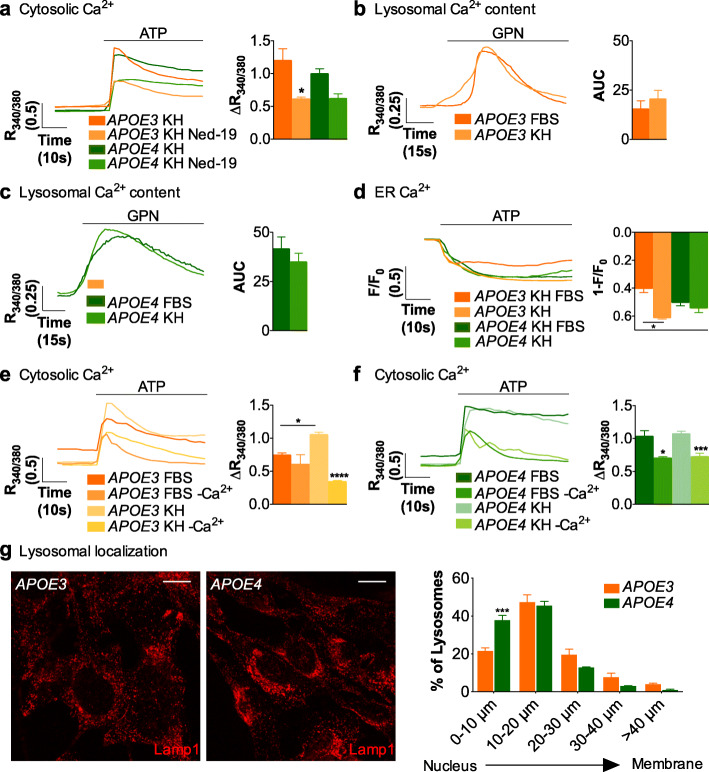
Loss of lipid-dependent coordination of Ca^2+^ fluxes across plasma and lysosomal membranes in *APOE4* immortalized astrocytes. **a** Representative traces and quantification of the magnitude of ATP- triggered intracellular Ca^2+^ responses in cells treated with DMSO or with 100 μM Ned 19 for 20 min in KH medium (*N* = 3). **b**, **c** Representative traces and quantification of lysosomal Ca^2+^ content measured as AUC after 200 μM GPN addition to (**b**) *APOE3* and (**c**) *APOE4* astrocytes in KH medium, as compared to DMEM medium supplemented with FBS (FBS) (*N* = 3) (FBS-related results were presented in Fig. 3); **d** Representative traces and quantification of Ca^2+^ ER release in 100 μM ATP-stimulated astrocytes transfected with G-CEPIA1er, and kept in KH medium supplemented or not with FBS (*N* = 4). **e**, **f** Representative traces and quantification of purinergic-induced Ca^2+^ responses in (**e**) *APOE3* and (**f**) *APOE4* astrocytes in KH medium supplemented with 10% of FBS with or without Ca^2+^/1 mM EGTA for 10 min, and KH medium with Ca^2+^ or without Ca^2+^/500 μM EGTA for 1 min. **g** Representative images from Lamp1 immunofluorescence of both cell lines and quantification of the percentage of lysosomes in different ranges of distance, the 0 value being the nucleus in each cell. (*N* = 3, 30 to 40 cells quantified). Scale bar represents 15 μm. One-way ANOVA was used in sections **a**, **d**, **e** and **f**, unpaired parametric T-test in **b** and **c**, and two-way ANOVA in **g**. *p* < 0.05 (*), *p* < 0.001 (***), *p* < 0.0001 (****)

Since the core of Ca^2+^-signaling dysregulation in *APOE4* astrocytes lies in acidic organelles, is Ca^2+^ homeostasis in these organelles the target of extracellular lipids in *APOE3* astrocytes? The finding that the increase in ATP-induced Ca^2+^ signals in *APOE3* cells in the absence of lipids/lipoproteins (KH media) was abrogated by Ned-19, the inhibitor of Ca^2+^ release from acidic stores (Fig. 7a) might indicate that extracellular lipids decrease Ca^2+^ levels inside such organelles. Surprisingly, measurement of Ca^2+^ stored inside acidic organelles using the GPN-elicited depletion showed no increased Ca^2+^ loading in *APOE3* cells kept in KH (Fig. 7b) compared to *APOE3* astrocytes kept in the presence of FBS, whereas, as expected, no difference was observed between the two conditions in *APOE4* astrocytes (Fig. 7c). An explanation is that, in the absence of extracellular lipids, the release of Ca^2+^ from lysosomes in *APOE3* astrocytes is amplified by the activation of other Ca^2+^ signaling mechanisms. In fact, the amplification originates in part, from Ca^2+^ released from the ER, because the Ca^2+^ content in the ER was 1.5-fold greater in *APOE3* cells kept in KH than in DMEM/FBS, as recorded with the Ca^2+^ probe CEPIA1er (Fig. 7d). The same amount of Ca^2+^ was released from the ER in *APOE4* astrocytes kept in the two media, in agreement with the observation that Ca^2+^ signals are independent of extracellular lipid concentrations in this genotype (Fig. 7d). Another source of Ca^2+^ is the extracellular Ca^2+^ entry, because the blockage of extracellular Ca^2+^ with the cell-impermeable Ca^2+^ chelator EGTA greatly reduced (by 3.3 times) ATP-induced Ca^2+^ responses in *APOE3* astrocytes kept in KH without lipoproteins, but not in *APOE3* astrocytes kept in KH with FBS (Fig. 7e). In contrast, EGTA reduced ATP-induced Ca^2+^ responses in *APOE4* astrocytes to the same extent in the presence or absence of FBS (Fig. 7f). Thus, the data support the idea that extracellular Ca^2+^ entry is secondary to lysosomal Ca^2+^ release, and that it is modulated by extracellular lipids in *APOE3* but not in *APOE4* astrocytes. We next sought morphological support for this idea by studying the localization of acidic stores inside astrocytes. Again, we resorted to Lamp1 immunostaining to be able to use confocal microscopy. Lysosome distribution was abnormal in *APOE4* cells (Fig. 7g); that is, a greater number of Lamp1-positive organelles accumulated near the nucleus in *APOE4* astrocytes than in *APOE3* astrocytes. Specifically, 37% of lysosomes are placed at 10 μm from the center of the nucleus in *APOE4* cells, as compared to 21% in *APOE3* astrocytes. Plausibly, the altered localization may result in changes in the coupling of such organelles with plasma-membrane channels.

In summary, in *APOE3* astrocytes, activation of extracellular Ca^2+^ entry secondary to intracellular Ca^2+^ mobilization underlies the greater Ca^2+^ responses induced by purinergic receptors in the absence of extracellular lipoproteins. This coordination of signaling pathways does not take place in the presence of lipoproteins, suggesting, again, that lipids have the capacity to change intracellular Ca^2+^ fluxes in *APOE3* astrocytes. By contrast, *APOE4* astrocytes present a higher content of lysosomal Ca^2+^, but appear to have lost the capacity to have Ca^2+^ fluxes regulated by lipids.

### Lipidomics reveals distinct lipid composition in lysosomal and whole-cell membranes from *APOE3* and *APOE4* astrocytes

**Fig. 8:**
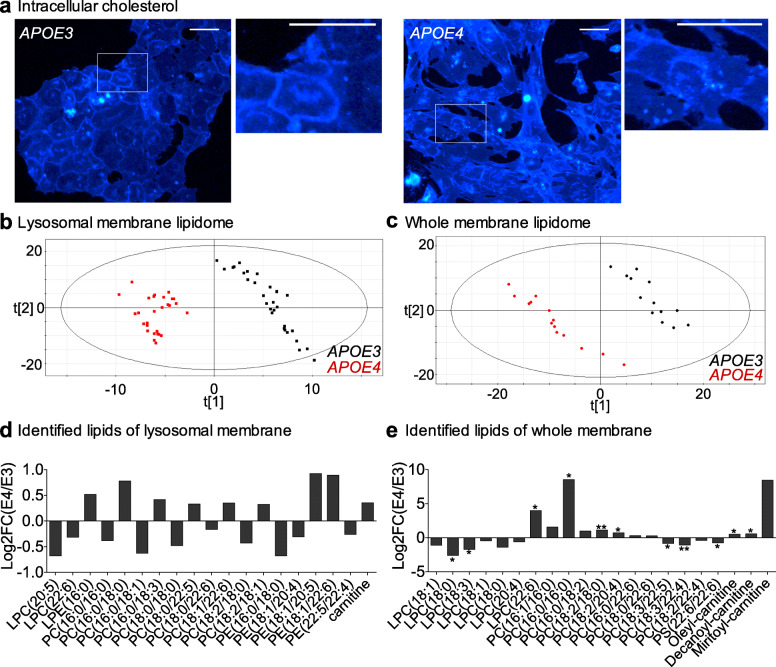
Different lipid profiles in lysosomal and whole-cell membranes from *APOE4* vs *APOE3* astrocytes. **a** Cholesterol accumulation visualized by Filipin III staining in immortalized *APOE3* and *APOE4* astrocytes (*N* = 2). Scale bar represents 100 μm. The white squares are amplified in the top right images. **b**, **c** PLS-DA analysis of lysosomal (**b**) and whole-cell membranes (**c**) from *APOE3* and *APOE4* immortalized astrocytes. Each dot is an individual sample. **d**, **e** Representation of changes of lipids with VIP > 1 identified in lysosome (**d**) and whole-cell (**e**) membranes as logarithm base 2 of fold changes of intensities in *APOE4* vs *APOE3*. Positive values indicate increase and negative values decrease in *APOE4* vs *APOE3* cells. q < 0.05 (*), q < 0.01 (**), multi T-test analysis corrected by false discovery rate (FDR)

We posited that lysosomal dysregulation and the refractoriness to lipid-based modulation in *APOE4* astrocytes might be caused by altered lipid trafficking and homeostasis due to *APOE4* malfunction—as concluded in a previous section. Since ApoE is a major cholesterol carrier in the brain, we studied cellular cholesterol distribution with filipin staining. We found aberrant intracellular distribution of cholesterol in *APOE4* astrocytes, which presented more cholesterol in intracellular clumps, and less in plasma membrane, than *APOE3* cells (Fig. 8a). This finding points to impaired cholesterol efflux in *APOE4* cells. We also carried untargeted lipidomics because we reasoned that impaired lipid trafficking would leave its mark on astrocyte membranes, such that the profiling of membrane lipids would provide information about lipid dyshomeostasis in *APOE4* astrocytes. To determine whether *APOE4*-mediated changes were specific to lysosomes, we performed lipidomics in lysosomal and whole-membranes of *APOE3* and *APOE4* immortalized astrocytes, since, according to a lipid map of the mammalian cell, organelles present distinct lipid compositions [66]. The multivariate analysis PLS-DA revealed that the lipids of lysosomal membranes (Fig. 8b) and whole-membranes (Fig. 8c) clustered independently in *APOE3* and *APOE4* genotypes. The predictive accuracy of the analysis was robust, as the Q^2^ and R_2_Y scores were 0.616 and 0.986 for lysosome lipidome, and 0.841 and 0.995 for whole-membranes. The PLS-DA analysis thus confirms that *APOE* genotype influences membrane lipid composition in astrocytes.

In order to identify which lipids contributed more to the differential group clustering, we used the Variable Importance in the Projection (VIP), such that lipids with VIP > 1 were the ones with greater weight on the group change. In lysosomal membranes, there were 35 lipids with VIP > 1 in the *APOE4* vs *APOE3*. ANOVA analysis with a Tukey correction post-test of the intensity (peak values) of these 35 metabolites revealed significant differences due to the *APOE* phenotype with a *p*-value < 0.05, confirming, again, that expression of *APOE4* alters the lipidome of lysosomes. We then proceeded to identification of the particular metabolites and calculation of their fold change (FC) in *APOE4* vs *APOE3* astrocytes. We could identify 19 lipids: 10 phosphatidylcholines (6 increased, 4 decreased), 5 phosphatidylethanolamine (2 increased, 3 decreased), 2 lysophosphatidylcholine (2 decreased), 1 lysophosphatidylethanolamine (decreased), and 1 carnitine (increased) (Fig. 8d). However, multi t-test analysis corrected by a false discovery rate (FDR) of these 19 FC values gave no statistically significant differences (q-value < 0.05). This suggests that joint changes in the contents of lipids with VIP > 1 rather than particular lipids account for the segregation of lipidomes from *APOE3* and *APOE4* lysosomes.

In whole-membranes, 41 metabolites had a VIP > 1, comparing *APOE4* vs *APOE3* astrocytes. ANOVA analysis with a Tukey correction post-test of the intensities of these 41 metabolites confirmed significant differences due to the *APOE* phenotype with a *p*-value < 0.01, in agreement with the previous PLS-DA analysis. Twety-one lipids were identified according to their m/z, and their FC in *APOE4* vs *APOE3* calculated (Fig. 8e). A multi T-test statistical analysis corrected with FDR, showed that 11 of these metabolites were significantly different (q-value < 0.05): 3 lysophosphatidylcholine (1 increased, 2 decreased), 5 phosphatidylcholines (3 increased, 2 decreased), 1 phosphatidylserine (decreased) and 2 carnitines (increased). In short, a general trend is that carnitines and phosphatidylcholines are more abundant in *APOE4* astrocytes, whereas *APOE3* cells are richer in lysophospholipids.

Overall, this is the first demonstration that the expression of *APOE4* changes the lysosomal and cellular lipidomes in astrocytes, supporting a link between altered Ca^2+^ fluxes and lipid dyshomeostasis. It is worth noting that the different intracellular distribution of cholesterol in *APOE3* and *APOE4* astrocytes is not due to differences in cholesterol contents between the two genotypes, as the VIP for cholesterol was consistently lower than 1 in the lipidomes (data not shown).

## Discussion

**Fig. 9:**
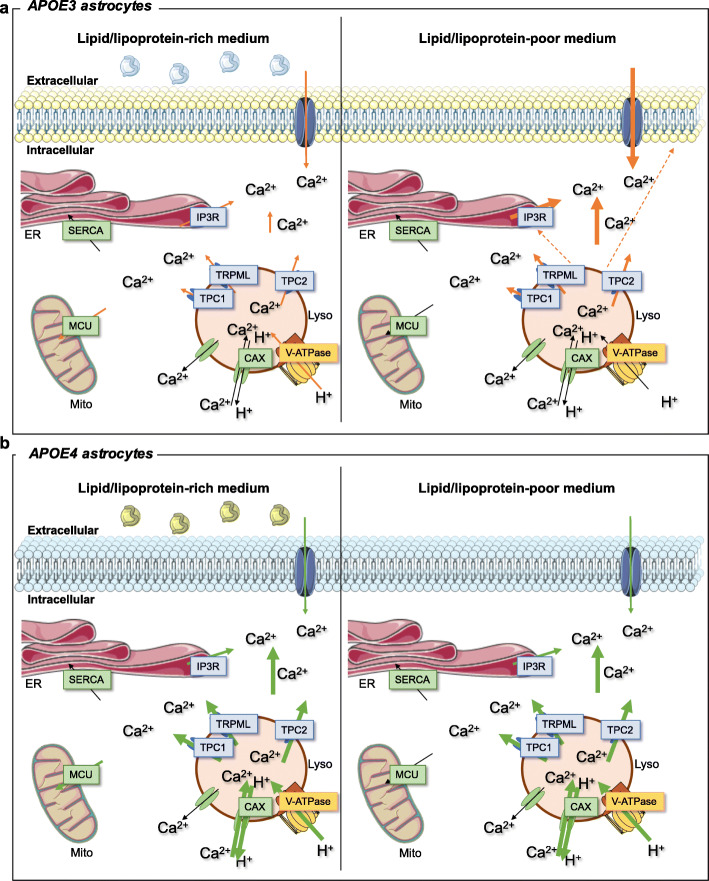
Summary of the Ca^2+^ signaling alterations in *APOE4* astrocytes. a) Ca^2+^ signaling pathway of *APOE3* (**a**) and *APOE4* (**b**) astrocytes in the presence (left) or absence (right) of extracellular lipids. The name of  channels and receptors that increase cytosolic Ca^2+^ are in blue rectangles, whereas pathways decreasing cytosolic Ca^2+^ are in green rectangles. The size of the arrows indicates if the process is increased with respect to *APOE3* astrocytes in the presence of extracellular lipids. Plasma-membrane lipids and lipoproteins are shown in different colors in *APOE3* and *APOE4* astrocytes to reflect their different lipid composition. Organelle and plasmatic membrane graphs were obtained from Smart Servier Medical Art (https://smart.servier.com/)

The study has two main general findings. First, in immortalized mouse astrocytes expressing human *APOE3* and *APOE4* we found that *APOE4*, in comparison to *APOE3*, increases receptor-induced Ca^2+^ responses due to increased release of Ca^2+^ from acidic organelles, which integrate lysosomes and related organelles. Further, *APOE4*-expressing astrocytes present distinct lipid profiles and are refractory to Ca^2+^-signaling regulation by lipids (model in Fig. 9). Second, Ca^2+^ hyperactivity associated with the *APOE4* allele was also found ex vivo in astrocytes from targeted replacement male mice, but not in females, whose astrocytes showed increased Ca^2+^ responses in *APOE3* mice, matching those in *APOE4* mice. Below we discuss the possible links between dysregulation of Ca^2+^ signaling, lipid signaling and lipid homeostasis in astrocytes, and the implications in neurodegenerative diseases in which *APOE4* is a risk factor in both men and women.

Mechanistically, a key finding is that the pH of acidic organelles is similar in immortalized *APOE4* and *APOE3* astrocytes, despite greater expression and activity of the lysosomal H^+^ pump V-ATPase, supporting the idea that the pH is maintained due to an antiparallel transport of H^+^ and Ca^2+^ that extrudes H^+^ and takes up Ca^2+^ via CAX. Thus, it is plausible that CAX expression and/or activity are increased in *APOE4* astrocytes, too. In consequence, *APOE4* acidic stores have greater Ca^2+^ content, and hence release more Ca^2+^ upon purinergic activation. Coordination between intracellular signaling pathways translates the increased Ca^2+^ mobilization from acidic vesicles to an increased Ca^2+^ released from ER, and a higher Ca^2+^ uptake into the mitochondria. In accordance with most studies in the field [36–38, 51, 58–60, 67–69], we considered this heterogeneous population of acidic vesicles as a whole, but we cannot rule out specific differential traits in a particular kind of acidic vesicle. For example, a recent report described differences of pH between the endolysosomal systems of *APOE3* and *APOE4* astrocytes [70], suggesting that greater pH differences than that detected in our study may exist between genotypes, depending on the compartment analyzed.

It is worth stressing that *APOE4* expression not only alters Ca^2+^ uptake and mobilization from acidic stores, but also promotes general dysregulation of these organelles in astrocytes, including higher perinuclear lysosome localization and changes in the overall lysosomal lipidome. The intracellular localization of lysosomes, which is determined by factors such as phospholipids and cholesterol, is linked to many of their functions and pathology [71, 72], although such studies have only been performed in cultured cells, plausibly due to the difficulty of tracking the tortuous intracellular distribution of the endolysosomal system in whole tissues. Specifically, our study confirms the previous observation that aberrant accumulation of cholesterol conditions promotes perinuclear clustering of lysosomes [71]. On the other hand, our results showing aberrant intracellular cholesterol accumulation, together with alterations of phospholipid composition in cellular membranes in *APOE4* astrocytes, point to dysregulation of the formation, internalization, and degradation of lipoproteins, processes in which lysosomes and related acidic vesicles participate [73]. Moreover, alterations of cholesterol trafficking and intracellular cholesterol accumulation have been linked to impaired autophagy [74]. Accordingly, impaired autophagy [75] and reduced lysosome-dependent amyloid degradation [76] have been reported in immortalized *APOE4* astrocytes, and in astrocytes derived from *APOE4* iPSC, respectively. Transcriptome analyses of whole brains of aged *APOE4* targeted replacement mice also revealed dysregulated expression of genes related to the endolysosomal system, although the contributions of the different cellular types were not studied [77]. Dysregulation of Ca^2+^ homeostasis might be a cause rather than a consequence of lysosomal dysfunction, as there is evidence supporting Ca^2+^ release from acidic stores controlling endolysosomal trafficking and autophagy [67, 78]. Moreover, in astrocytes, it has been reported that NAADP-induced Ca^2+^ release from lysosome-like organelles increases autophagic markers [79], and inhibits the fusion of the autophagosome with lysosomes, thus arresting the autophagic fluxes [69].

An unexpected discovery of this study is that lipoproteins modulate the magnitude of ATP-induced cytosolic Ca^2+^ responses in *APOE3* astrocytes by changing the interplay of Ca^2+^ signaling and fluxes among organelles and the plasma membrane. The observation that the down-regulation of purinergic-induced Ca^2+^ responses in the presence of lipids is quick and reversible rules out the implication of gene expression and down-regulation of purinergic receptors. Rather, the phenomenon supports the emerging notion of lipid-mediated control of Ca^2+^ channels. Precedents are the activation by lysophosphatidylcholine of astrocytic extracellular Ca^2+^ entry [80], and the regulation by lipids of some of the channels responsible for SOCE. Specifically, cholesterol regulates TRPC1 in neutrophils [81] and STIM in pulmonary endothelial cells [82], whereas phosphoinositides regulate TRPC3,6,7 channels in numerous cell types [83]. Recently, very-low-density lipoproteins have been shown to inhibit STIM in atrial myocytes [84]; this study and ours are the first to report lipoproteins regulating Ca^2+^ signaling. It is worth stressing that the all-or-nothing experimental design consisting in testing the effects of media with and without lipoproteins allowed us to obtain proof of concept that lipoproteins modulate Ca^2+^ excitability in astrocytes, but in physiological settings, Ca^2+^ signaling in astrocytes is, plausibly, modulated by subtle changes in brain lipid contents.

Importantly, lipoprotein-mediated regulation of purinergic Ca^2+^ signaling is lost in *APOE4* astrocytes. Several factors could explain this finding. First, the accumulation of lysosomes around the nucleus may uncouple the lysosomal Ca^2+^ release and SOCE. Second, the entry of extracellular Ca^2+^ triggered by low levels of Ca^2+^ inside acidic vesicles requires the TPC2 channels of acidic vesicles, which are less expressed in *APOE4* than in *APOE3* astrocytes. Third, Ca^2+^ inside the acidic stores is higher in *APOE4* astrocytes compared to *APOE3* cells; hence SOCE mechanisms may not be triggered after purinergic-induced lysosomal Ca^2+^ release. Fourth, *APOE4* astrocytes may be devoid of the right concentration of lipids to modulate lysosomal and Ca^2+^ entry channels, as they have different lysosomal and cellular lipidomes compared to *APOE3* cells. Specifically, the decreased contents of lysophosphatidylcholine species in *APOE4* vs *APOE3* cellular membranes may explain the uncoupling of Ca^2+^ fluxes in *APOE4* astrocytes, for these lipids activate SOCE [80], and the cation TRPV2 channel [85] in astrocytes. Moreover, the increase in phosphatidylcholines in *APOE4* lysosomes is consistent with the observed potentiation of NAADP-mediated Ca^2+^ release, since, as noted, phosphatidylcholines stimulate this pathway [65]. Finally, the decrease in plasma membrane cholesterol in *APOE4* astrocytes compared to *APOE3* cells may underlie the reduced SOCE activation, because SOCE requires cholesterol in different cell types [81, 82, 86]. Cholesterol alterations have been also reported in human *APOE4*-iPSC derived astrocytes [76], and in brains from 12-month-old *APOE4* mice [87]. Interestingly, cholesterol synthesis is decreased in astrocytes upon aging [88], which might exacerbate *APOE4*-elicited lipid dyshomeostasis and Ca^2+^ signaling.

Remarkably, the differences in Ca^2+^ signaling between *APOE4* and *APOE3* astrocytes were also detected ex vivo in 9–12-week-old male but not in female mice, suggesting that the *APOE* allele affects astrocyte excitability in a sex-dependent manner. The present study thus adds to the increasing evidence of complex interactions between *APOE* genotype and sex, as shown, for example, in lipid-related metabolic variations [89], cerebrovascular pathology [90], and tau levels in the cerebrospinal fluid [91]. The following scenarios might explain the sex bias in astrocyte excitability in different *APOE* alleles. First, Ca^2+^ signals in female astrocytes might be by default higher, regardless of the *APOE* allele. Along these lines, a recent study showed greater estradiol-induced Ca^2+^ signals in astrocytes from female mice than from male astrocytes [92]. Second, the down-regulation of Ca^2+^-based excitability by lipids observed in immortalized *APOE3* astrocytes might not occur in females. Note that ex-vivo Ca^2+^ responses in female mice resemble Ca^2+^ responses in immortalized astrocytes in the absence of lipoproteins. Emerging evidence indeed points to a distinct impact of *APOE* genotype on brain lipid metabolism [76, 87] with a sex bias [93]. In the latter study, lipid clustering by principal component analysis of cortical lipidomes unravels sample segregation by sex in *APOE3* but not in *APOE4* 16 month-old mice [93]. That is, sex differences were observed in *APOE3* but not in *APOE4* mice, as in our study. Specifically, there was a trend for greater concentrations of phosphatidylcholines and lysophosphatidylcholines in *APOE3* male than in *APOE3* female mice [93]. Taking together these and our results, it is plausible that the interplay of sex, *APOE* genotype and age differently shapes the composition of lipid *milieus* in male and females through life, resulting in distinct astrocytic Ca^2+^ responses.

Whatever the case, our study supports that immortalized astrocytes from human *APOE* replacement mice may be a model to understand *APOE4* pathology in males. The fact that Ca^2+^-based astrocyte excitability controls neural functions [94] lends credence to the hypothesis that *APOE4*-elicited dysregulation of Ca^2+^ fluxes in astrocytes contributes to the impairment of brain activity and metabolism in the healthy brain, as repeatedly reported in humans [4–6, 8], although, of note, no sex-based stratification existed in these studies. It is worth noting that the differences in *APOE* genotype were observed in 10-week old male mice, pointing to early detrimental actions of *APOE4* in brain, not surprisingly so, for *APOE* alleles are acquired at conception. This is important because, although metabolic alterations and distinct patterns of brain activity have been reported in young humans harboring *APOE4* [95, 96], and olfactory-memory impairment exists in 6 month-old *APOE4* mice [13, 89], most studies with human *APOE4* gene targeted replacement of murine *APOE* have been conducted with aged mice, and occasionally, middle-aged (over 10 months) rodents [97].

We emphasize that the *APOE4* phenotype observed in the aforementioned studies, as well as in our study, occurs in the absence of LOAD pathology, although it may render brains more vulnerable to age-dependent ailments. Thus, a wealth of data suggests that *APOE4* exacerbates the impairment of Aβ processing and clearance caused by ApoE, leading to increased accumulation, and hence aggregation, of Aβ in the brain [70]. In addition, ApoE4 has recently been reported to potentiate Tau-mediated neurodegeneration independently of Aβ [24]. However, it is increasingly more recognized that alterations in lysosomal functions [98], lipid homeostasis [99], and Ca^2+^ signaling play a role in LOAD, too. The Ca^2+^ hypothesis in LOAD contends that aberrant Ca^2+^ responses in neurons associated with Aβ, Tau, and the glutamatergic system underlie cognitive impairment [100]. In addition, Ca^2+^ signaling is profoundly dysregulated in astrocytes in animal models of AD [39, 101], mainly due to aberrant activation of purinergic receptors [56]. Our results support the notion that *APOE4* may exacerbate the dysregulation of Ca^2+^ signaling in male astrocytes in LOAD owing to lysosomal dysfunction caused by lipid dyshomeostasis. Dysregulation of astrocyte excitability in LOAD may, in turn, contribute to neural-circuit hyperactivity independently of Aβ and Tau pathologies [102], supporting astrocytic-lysosome targeted therapies in LOAD, at least in male patients.

An outstanding question in *APOE4*-targeted therapeutics is whether the mutated domain renders ApoE4 toxic, or less efficient, than ApoE3 and ApoE2. Alternatively, since ApoE is lower in cerebrospinal fluid (CSF) of *APOE4* individuals [62], and in plasma, CSF, and brain tissue of *APOE3* and *APOE4* as compared with *APOE2* targeted replacement mice [103], is it the problem that ApoE4 is less abundant? The answers to these questions are important because they will determine whether therapeutic strategies should be aimed to increase or decrease ApoE production, or to replace *APOE4* with *APOE3* or APOE2. It seems unlikely that the effects of *APOE4* on lysosomal-related Ca^2+^ fluxes are due to decreased expression of *APOE4*, because *APOE4* over-expression did not revert Ca^2+^ responses to the levels observed in *APOE3* cells. It is also unlikely that the *APOE4* phenotypes are caused by direct interaction between ApoE4 and V-ATPase and/or NAADP receptors in the acidic organelles, as shown for APOD, another brain apolipoprotein that participates in vesicle-mediated astrocyte-to-neuron communication [104] and prevents lysosomal membrane permeabilization [51], given that our immunocytochemistry showed scarce ApoE in astrocytic lysosomes. Rather, the data support malfunction of ApoE4, which could be rescued by over-expression of *APOE3*, as shown here, and perhaps *APOE2* [50]. Whether drugs designed to increase ApoE4 lipidation in order to enhance the capacity of the lipoprotein to carry cholesterol [105] would restore intracellular lipid dyshomeostasis in astrocytes is an open question, since the mechanisms whereby the *APOE* allele modifies lipid contents and distribution in astrocyte membranes remain to be explored. Alternatively, because *APOE4* exacerbates ApoE-mediated Aβ aggregation [70] and Tau pathology [24], ApoE removal is being pursued by immunotherapy [106]. However, considering the pleiotropic functions of *APOE* as a lipid carrier between cells, and plausibly inside cells, chronic removal of ApoE may have secondary effects. All in all, development of *APOE4*-specific therapeutics is in order. To this end, better understanding of the basic biology of *APOE*, and of the particulars of *APOE4* action and sex bias independently of Aβ and Tau, are necessary. Overall, our findings that *APOE4* per se disrupts the lipid-based regulation of Ca^+ 2^ fluxes in astrocytes due to lysosomal dysregulation supports the notion that astrocyte dysfunction contributes to *APOE4* pathology in neurodegeneration.

## Conclusions

Taking together ex vivo and in vitro data, we conclude that *APOE4* malfunction augments Ca^2+^-based excitability in astrocytes due to dysregulation of calcium fluxes in and out the lysosome, associated with lipid dyshomeostasis, and that the phenomenon might be male-associated. One implication of the study is that it supports the use of therapies aimed to restoring lysosomal dysfunction in astrocytes, including targeted overexpression of APOE3 or APOE2, in order to halt the accelerated progression of LOAD in male APOE4 carriers. Since Ca^2+^ signaling is central to the regulation of neural circuits by astrocytes, another implication of the study is that the therapeutic correction of astrocyte excitability might reverse the neural-circuit hyperactivity observed in APOE4-harboring humans and mice in the absence of Aβ and Tau pathologies. Finally, clarifying the sex bias in the efficacy of APOE4 targeted therapeutics is a must, for the mechanisms underlying APOE4 pathology might differ in males and females.

## Data Availability

Not applicable.

## Abbreviations

APOE: Apolipoprotein E
ACSF: Artificial cerebrospinal fluid medium
AUC: Area under the curve
CAX: Ca2+/H+ exchanger
CSF: Cerebrospinal fluid
ER: Endoplasmic reticulum
FBS: Fetal bovine serum
FC: Fold change
GECI: Genetically encoded Ca^2+^ indicators
GPN: Glycyl-L-phenylalanine 2-naphthylamide
h: Hours
IP3: Inositol 1,4,5-triphosphate
KH: Krebs medium
Lipo: Lipofectamine
LOAD: Late-onset Alzheimer’s disease
LPC: Lysophosphatidylcholine
LPE: Lysophosphatidylethanolamine
LTP: Long-term potentiation
min: Minutes
NAADP: Nicotinic acid adenine dinucleotide phosphate
NMDG: N-methyl-D-glucamine
PC: Phosphatidylcholine
PE: Phosphatidylethanolamine
PS: Phosphatidylserine
ROI: Region of interest
s: Seconds
Sc: Scramble
SOCE: Store-operated Ca^2+^ entry
SR101: Sulforhodamine
Tpc1: Two-pore channels 1
Tpc2: Two-pore channels 2
Trpml: Transient receptor potential mucolipin
TTX: Tetrodotoxin
VIP: Variable importance in projection
Vs: Versus
